# LINC00707 impairs the Natural Killer cell antitumour activity in hepatocellular carcinoma through decreasing YTHDF2 stability

**DOI:** 10.1111/jcmm.18106

**Published:** 2024-01-18

**Authors:** Mingwei Wei, Libai Lu, Jiasheng Ma, Zongjiang Luo, Xijuan Tan, Jianchu Wang

**Affiliations:** ^1^ Department of General Surgery The First Affiliated Hospital of Jinan University Guangzhou China; ^2^ Department of Hepatobiliary and Pancreatic Surgery, Baidong Hospital Affiliated Hospital of Youjiang Medical University for Nationalities Baise China; ^3^ Guangxi Clinical Medical Research Center for Hepatobiliary Diseases the Affiliated Hospital of Youjiang Medical University for Nationalities Baise China

**Keywords:** hepatocellular carcinoma, LINC00707, Natural Killer cell, YTHDF2

## Abstract

Hepatocellular carcinoma (HCC) is the fifth most frequently diagnosed cancer and ranks third in cancer‐related fatalities. The recognized involvement of long noncoding RNAs (lncRNAs) in several cancer types, including HCC, inspired this study to explore a novel lncRNA's functional importance in the progression of HCC. To achieve this, lncRNA microarray analysis was conducted on three distinct sets of HCC tissues, revealing LINC00707 as the most significantly upregulated lncRNA. Further research into its biological functions has revealed that LINC00707 acts as an oncogene, driving HCC progression by enhancing the proliferation, migration and invasion of HCC cells. Mechanistic insights were provided, demonstrating that LINC00707 interacts with YTH N6‐methyladenosine RNA‐binding protein 2 (YTHDF2), thus facilitating the ubiquitination‐dependent degradation of the YTHDF2 protein. Furthermore, LINC00707 was found to influence the cytotoxicity of NK‐92MI cells against HCC cells through its interactions with YTHDF2. These findings significantly contribute to a deeper understanding of the role played by LINC00707 in the progression of HCC.

## 1 INTRODUCTION

On a global scale, hepatocellular carcinoma (HCC) stands at the fifth position in terms of cancer incidence and holds the third position for cancer‐related mortality.^1^, ^2^ Although there have been notable advancements in treatments such as surgery, radiofrequency ablation, transarterial chemoembolization, targeted therapy and emerging immunotherapies, there is still potential for improving the survival rates of HCC patients.^3^, ^4^, ^5^ Therefore, there is an urgent demand for a novel theoretical framework of biological markers to be utilized in clinical scenarios. This framework would aid in improving early diagnosis, facilitating the development of targeted therapies for HCC and ultimately elevating the survival prospects of individuals afflicted with HCC.

A long noncoding RNA (lncRNA) is characterized by its length exceeding 200 bp and its absence of significant protein‐coding capacity or by possessing only a limited ability for protein coding.^6^ LncRNAs play various molecular roles, including serving as host genes for microRNAs (miRNAs), hindering the binding of RNA and proteins to their intended targets and acting as molecular scaffolds that guide proteins towards specific chromosomal targets.^7^ The regulation of lncRNA expression within tumours is intricate and multifaceted. LncRNAs contribute to several pivotal cancer traits by engaging with biological macromolecules like DNA, mRNAs and proteins. In this way, they exert regulatory influence on the initiation and progression of tumours.^8^, ^9^ However, in comparison to messenger RNAs (mRNAs), lncRNAs exhibit relatively modest expression levels, diverse forms of generation, intricate structural attributes and noncoding characteristics. Additionally, they generally lack evolutionary conservation, leading to a substantial portion of lncRNA functions remaining unexplored.^10^ Furthermore, the precise epigenomic regulatory network of lncRNAs remains predominantly obscure. Research has indicated that, in the context of HCC, lncRNAs hold substantial significance. Aberrant expression of various proteins associated with HCC leads to interactions with lncRNAs, subsequently governing the cancer‐related characteristics.^11^ This underscores the considerable potential of lncRNAs as novel cancer markers and promising targets for therapeutic interventions.

The primary objective of this study was to unveil a previously undiscovered functional lncRNA associated with the advancement of HCC. Through an analysis of lncRNA microarrays on three sets of HCC tumour tissues and their corresponding adjacent normal tissues, it was discovered that LINC00707 displayed one of the most significant upregulations among the lncRNAs. The literature has previously demonstrated the role of LINC00707 in the progression of HCC.^12^, ^13^ However, the precise mechanism through which LINC00707 exacerbates the development of HCC remains incompletely understood. In this context, the current study discovered that LINC00707 is capable of binding to YTHDF2, subsequently influencing the cytotoxicity of Natural Killer (NK) cells against HCC cells. These findings provide a partial understanding of the role played by LINC00707 in HCC progression.

## 2 MATERIALS AND METHODS

### 2.1 Tissue sample and analysis

Between July 2021 and April 2022, three patients diagnosed with HCC were included in the study, resulting in six tissue samples comprising three HCC tissues and their corresponding adjacent noncancerous tissues. Before collection, all patients were not received any chemotherapy or radiotherapy. These samples were collected from Baidong Hospital, Affiliated Hospital of Youjiang Medical University for Nationalities in China. All tissues were utilized for transcriptome microarray profiling. Following collection, the clinical specimens were promptly frozen using RNAprotect tissue reagent from Qiagen, adhering to the manufacturer's recommended procedures. The study adhered to ethical guidelines and secured written informed consent from all participating patients. The research protocol obtained approval from the medical ethics committee of Baidong Hospital, which is the Affiliated Hospital of Youjiang Medical University for Nationalities. The Affymetrix Human Transcriptome Array 2.0 was employed to identify differentially expressed lncRNAs. The data analysis was carried out using the robust multichip analysis algorithm. Differentially expressed lncRNAs were identified using specific criteria: a fold change exceeding 1.2 and a *p*‐value below 0.05.

### 2.2 Cell culture and treatment

Various cell lines, including HL‐7702, Hep3B, SMMC‐7721, SK‐HEP‐1, Huh7 and SNU449, were cultured in DMEM obtained from HyClone. The culture medium was enriched with 10% fetal bovine serum (FBS) sourced from Gibco and 1% penicillin/streptomycin obtained from Sigma‐Aldrich. These cells were maintained in a humidified incubator at 37°C with 5% CO_2_.

Plasmids carrying short hairpin sequences against LINC00707 and YTHDF2, as well as full‐length YTHDF2 plasmids, were synthesized by GenePharma in Shanghai, China. These plasmids also included their respective non‐targeting sequences as negative controls (NC). The cells were first seeded into 24‐well plates and then transfected using Lipofectamine 3000 reagent along with Opti‐MEM I from Life Technologies Corporation, Carlsbad, CA. This procedure was carried out in accordance with the manufacturer's instructions. Stably transfected cells were subsequently selected using Geneticin (G418) acquired from Sigma‐Aldrich, St Louis, MO, USA.

### 2.3 RT‐qPCR

The total cellular RNA was extracted using the FastPure RNA Isolation Kit from Vazyme Biotech Co., Ltd. Subsequently, RNAs were converted into cDNA using a reverse‐transcription system kit from Takara. For RT‐qPCR analysis, ChamQ SYBR qPCR Master Mix (Low ROX Premixed) from Vazyme Biotech Co., Ltd was used, following the manufacturer's instructions. GAPDH was utilized as the internal control. Relative analysis was performed using the comparative CT (2^−ΔΔCT^) method, with each assay being independently replicated three times. The gene‐specific primers used in this study were as follows: LINC00707 (F: 5′‐TCACATCTGTGAAAAGAGTGCT‐3′; R: 5′‐CTGGACTGTGAGTACCAGGC‐3′), YTHDF2 (F: 5′‐AGCCCCACTTCCTACCAGATG‐3′; R: 5′‐TGAGAACTGTTATTTCCCCATGC‐3′), GAPDH (F: 5′‐GCACCGTCAAGGCTGAGAAC‐3′; R: 5′‐TGGTGAAGAC GCCAGTGGA‐3′).

### 2.4 Western blot

For protein analysis, 20 μg of protein was separated through SDS‐PAGE electrophoresis. PVDF membranes were utilized and blocked with 5% skimmed dry milk. Primary antibodies, including rabbit polyclonal to YTHDF2 and mouse monoclonal to GAPDH from Cell Signaling Technology in Danvers, MA, USA, were applied. After an overnight incubation at 4°C, the membranes were washed with TBST. The following day, the membranes underwent additional incubation with secondary antibodies. Finally, the immunoblots were visualized using an enhanced chemiluminescence reagent.

### 2.5 Fluorescence in situ hybridization (FISH)

Hep3B and SNU449 cells were seeded in 24‐well plates at a density of 3 × 10^4^ cells per well. Subsequently, the cells were fixed with 4% paraformaldehyde (PFA) for 15 min at room temperature. After fixation, they were permeabilized with 0.5% Triton X‐100 for 15 min at 4°C. Following this, the cells were exposed to probes and incubated at 55°C for 4 h. After incubation, the cells were washed for 5 min with 2× PBS and then treated with secondary antibodies conjugated with horseradish peroxidase (HRP) obtained from Jackson, West Grove, PA, USA. Finally, the cells were counterstained with DAPI, and images were captured using an Olympus confocal laser scanning microscope.

### 2.6 RNA pull‐down

The specific genetic sequences associated with LINC00707 were seamlessly integrated into the pcDNA3.1 vector, which included a meticulously designed segment for binding with YTHDF2. These carefully constructed plasmids, along with the Flag‐tagged YTHDF2 plasmid, were co‐transfected into the HCC cells under investigation. Subsequently, RNA molecules that interacted with YTHDF2 were immunoprecipitated with precision, utilizing Flag antibodies sourced from Proteintech. Following this step, we conducted pull‐down assays with the aid of advanced magnetic bead technology. The proteins firmly bound to these magnetic beads were accurately identified through western blot analysis, while the RNA molecules exhibiting enrichment were precisely quantified using state‐of‐the‐art RT‐qPCR techniques.

### 2.7 RNA immunoprecipitation (RIP)

Ago2‐binding RNA was extracted using the Magna RIP™ Kit from Millipore. Initially, cells were harvested and lysed with a specific lysis buffer. Subsequently, the resulting cell lysates were combined with RIP buffer containing magnetic beads. For binding to the beads, an Ago‐2 antibody from Abcam was utilized, with an anti‐IgG antibody from Abcam serving as the negative control. To further process the samples, DNase I and Proteinase K were applied for digestion. The RNA enriched during this procedure was subsequently converted into cDNA and subjected to analysis through RT‐qPCR.

### 2.8 Cell colony assay

Hep3B and SNU449 cells, previously transfected with the respective plasmids, were evenly distributed into separate wells of 6‐well plates, with each well containing 2 × 10^3^ cells. These plates were then placed in a sterile incubator, maintaining a temperature of 37°C for a period of 6 days. After this incubation period, the cell colonies were treated with a crystal violet staining solution. Subsequently, the stained colonies were meticulously observed and subjected to thorough analysis.

### 2.9 Transwell assay

In the migration assay, cells were carefully placed into the upper chamber of Transwell assay inserts featuring a membrane equipped with 8 mm pores. To facilitate migration, the upper chamber was filled with 200 μL of serum‐free DMEM. Following a defined incubation period, cells residing on the upper surface were meticulously stained using crystal violet. Following staining, the cells were systematically captured using a digital microscope.

Conversely, in the invasion assay, cells were thoughtfully seeded in the upper chamber, which featured a Matrigel‐coated membrane. An incubation period of 48 h allowed the cells to exhibit their invasive capabilities. Subsequently, the invasive capacity was meticulously assessed using the same protocol employed for the migration assay.

### 2.10 Lactate dehydrogenase (LDH) assay

To assess the cytotoxic effects of NK‐92MI cells against HCC cells, we employed the CytoTox 96® Non‐Radioactive Cytotoxicity Assay Kit (#G1780, Promega, Madison, WI, USA). In a nutshell, HCC cancer cells were initially subjected to transfection. After a 24‐h incubation period, NK‐92 MI cells were introduced into the culture and co‐cultured with the previously transfected HCC cells. Various ratios were tested, specifically 5:1, 10:1 and 20:1 (target cells: effector cells), during a 6‐h co‐culture period. Subsequently, the cell culture supernatants were meticulously collected to quantify the release of LDH.

### 2.11 ELISA

To quantify the protein levels of tumour necrosis factor‐alpha (TNF‐α) and interferon‐gamma (IFN‐γ) in the cell supernatants, we employed the IFN‐γ human ELISA Kit (#RK00015, ABclonal, MA, USA) and the TNF‐α human ELISA Kit (#KE00154, Proteintech, Rosemont, IL, USA). The assays were meticulously conducted, following the detailed instructions provided by the respective manufacturers.

### 2.12 Statistical analysis

Statistical analysis was performed using GraphPad Prism 6.0 software. Comparisons between two groups were conducted using the Student's *t*‐test, while for multiple group comparisons, a one‐way ANOVA was utilized. The significance level was established at a *p*‐value below 0.05.

## 3 RESULTS

### 3.1 LINC00707 is upregulated in HCC

**FIGURE 1:**
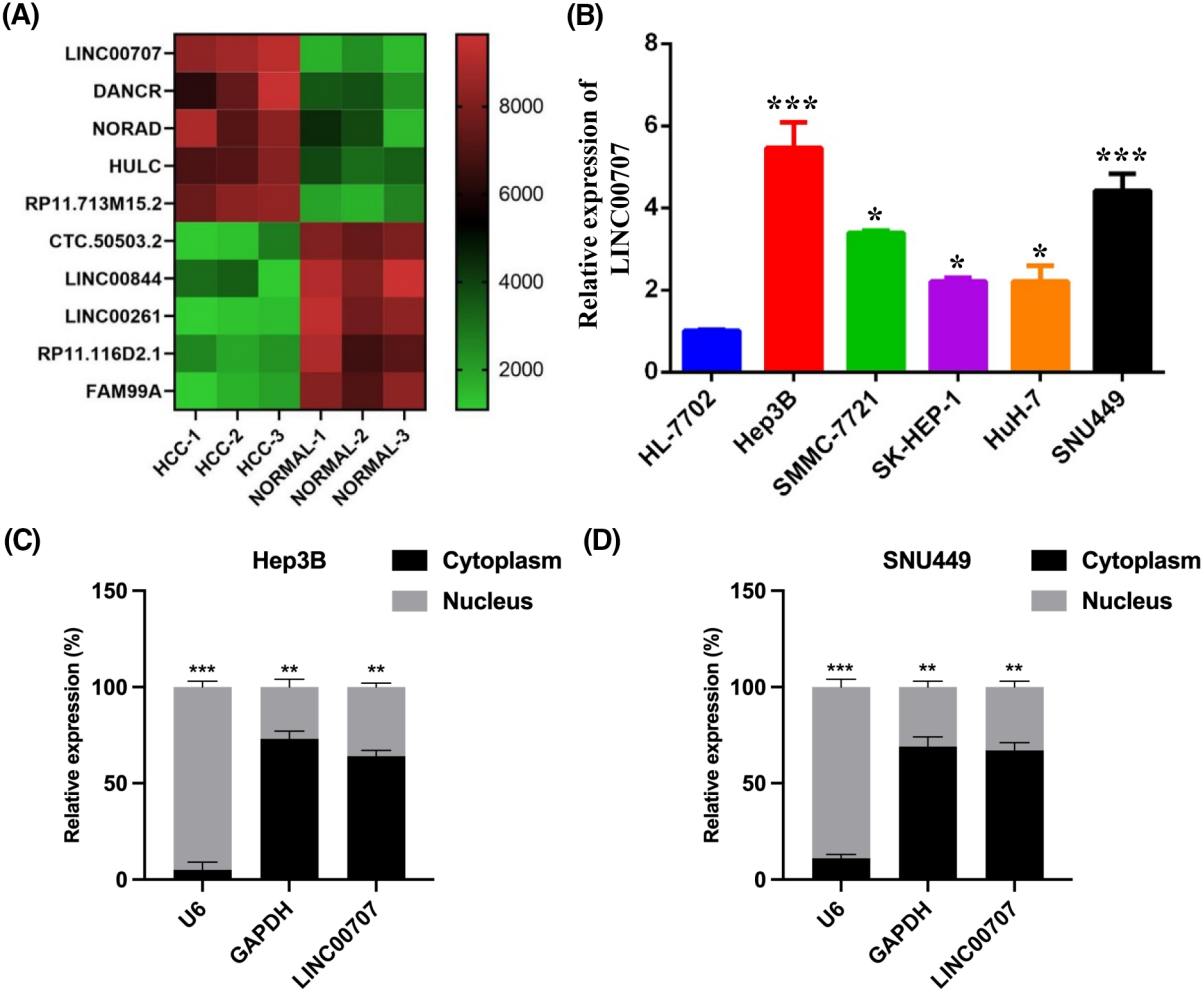
LINC00707 is upregulated in hepatocellular carcinoma (HCC). (A) Long noncoding RNA microarray analysis was performed on three pairs of HCC tumour or normal tissue samples; the most significantly dysregulated genes were presented as heatmap. (B) qRT‐PCR assay was conducted to evaluate the expression level in HCC cell lines (Hep3B, SMMC‐7721, SK‐HEP‐1, Huh‐7 and SNU449) and the normal cell line (HL‐7702). (C) Cellular distribution of LINC00707 in Hep3B cells. (D) Cellular distribution of LINC00707 in SNU449 cells. The experiments were performed three times. *p < 0.05, **p < 0.01, ***p < 0.001.

To explore the involvement of lncRNAs in the development of HCC, we conducted lncRNA microarray analysis on three pairs of HCC tumour tissues and adjacent normal tissues. Among the identified lncRNAs, LINC00707 exhibited the most significant upregulation in the tumour tissues (Figure 1A). Subsequent qRT‐PCR results demonstrated that LINC00707 was notably overexpressed in HCC cell lines, particularly in Hep3B and SNU449 cells, when compared to the normal HL‐7702 cell line (Figure 1B). Furthermore, we investigated the subcellular localization of LINC00707 within HCC cells and observed that LINC00707 predominantly resided in the cytoplasm of both Hep3B and SNU449 cells (Figure 1C,D).

### 3.2 LINC00707 knockdown attenuates HCC cell proliferation, migration and invasion

**FIGURE 2:**
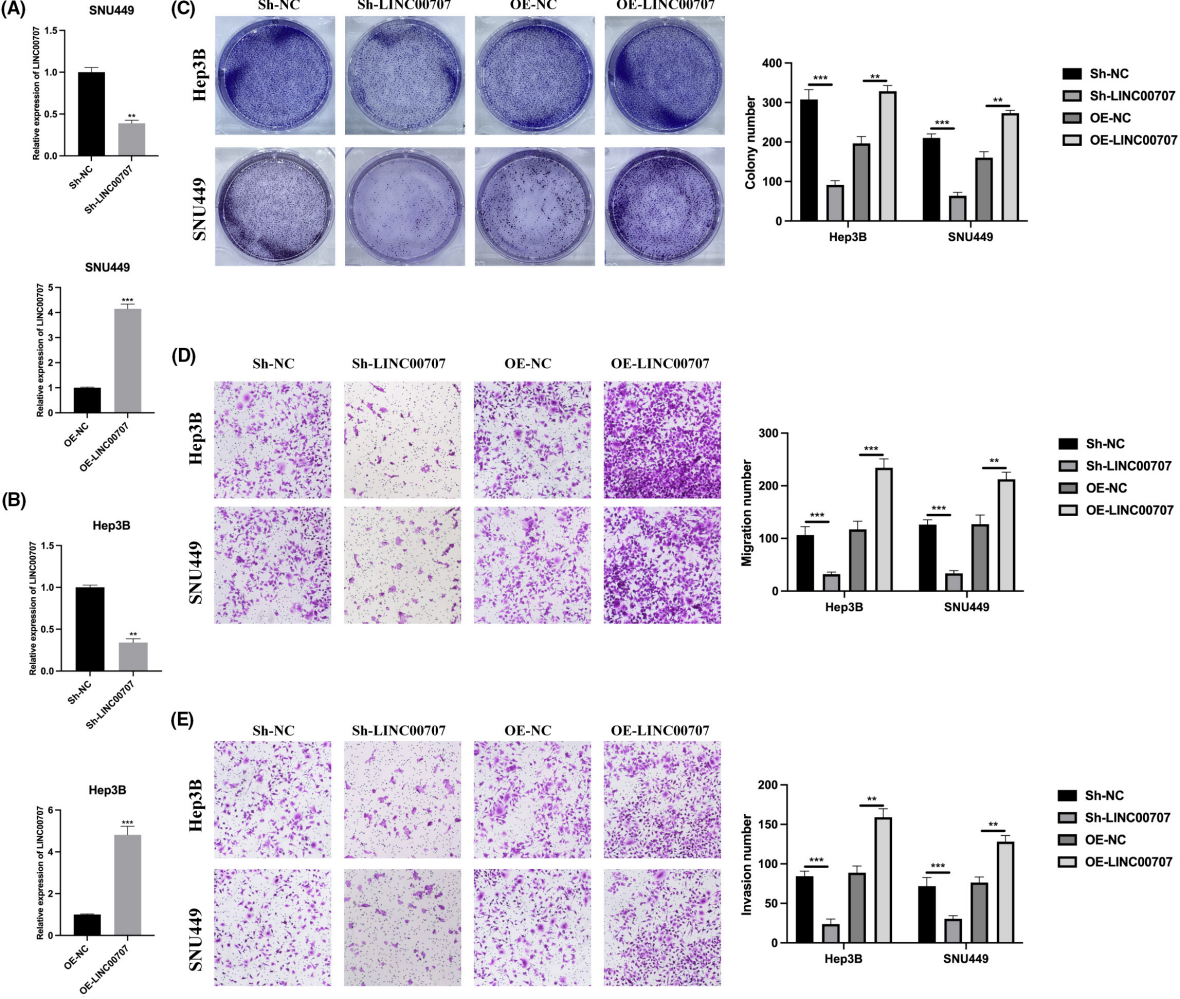
LINC00707 knockdown attenuates hepatocellular carcinoma cell proliferation, migration and invasion. (A, B) SNU449 and Hep3B cells were introduced with sh‐NC, sh‐LINC00707, OE‐NC and OE‐LINC00707, and LINC00707 expression was measured by qRT‐PCR. (C) Cell colony assay was conducted to evaluate cell proliferation level. (D) Transwell migration assay was used to detect cell migration ability. (E) Transwell invasion assay was utilized to elucidate cell invasion level. The experiments were performed three times. **p < 0.01, ***p < 0.001.

To elucidate the biological functions of LINC00707, we conducted experiments involving the modulation of LINC00707 expression in Hep3B and SNU449 cells, followed by the quantification of results via qRT‐PCR (Figure 2A,B). Subsequently, we performed a cell colony assay to evaluate the impact of LINC00707 on HCC proliferation. The findings revealed that downregulation of LINC00707 resulted in a decrease in the proliferation of HCC, while upregulation of LINC00707 led to an increase in proliferation (refer to Figure 2C). Moreover, similar trends were observed in transwell assays, where we assessed both migration (Figure 2D) and invasion (Figure 2E) capabilities. Specifically, downregulation of LINC00707 corresponded to decreased migration and invasion abilities in HCC cells, whereas upregulation of LINC00707 was associated with an enhancement in both migration and invasion capacities.

### 3.3 LINC00707 binds to YTHDF2

**FIGURE 3:**
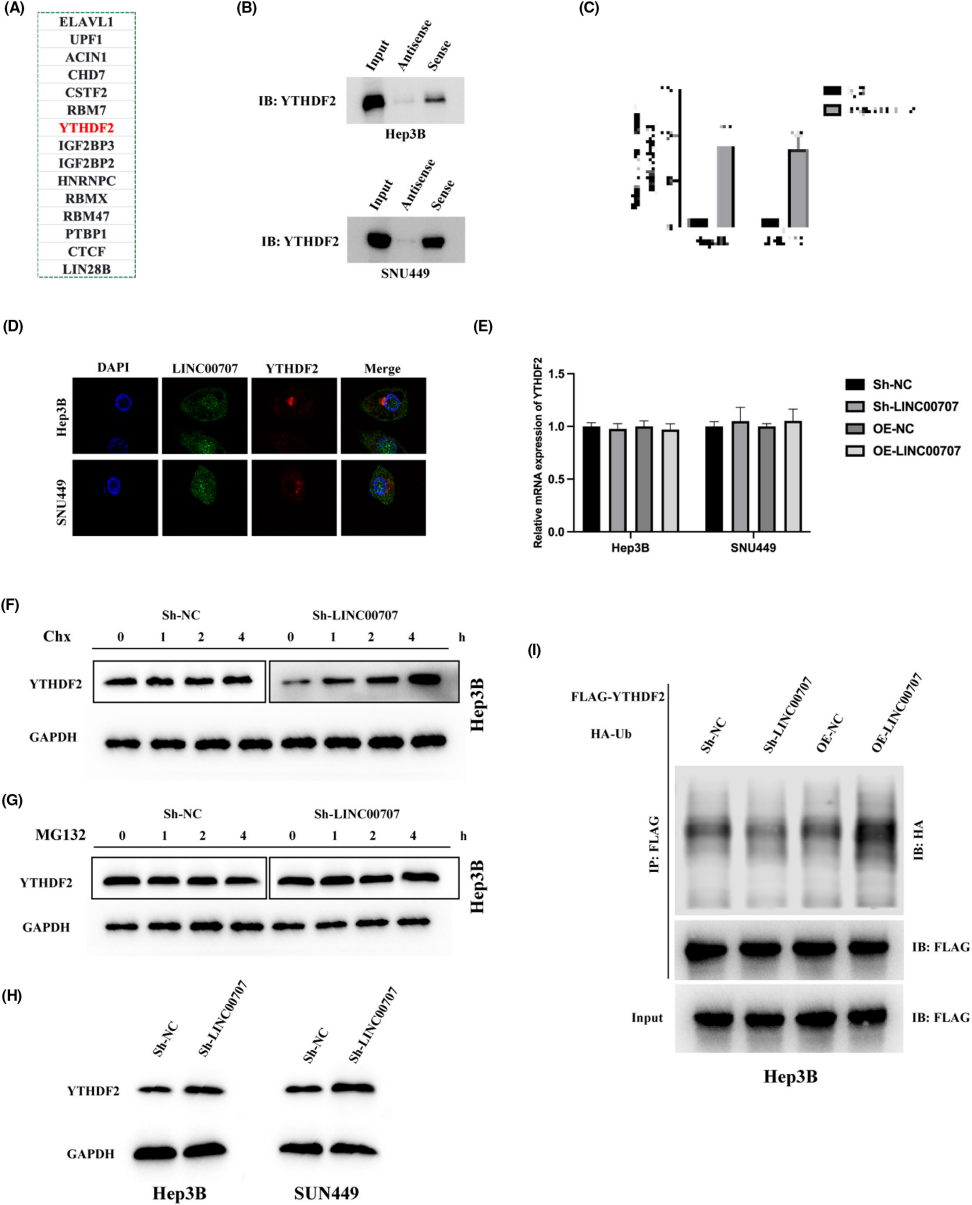
LINC00707 binding to YTHDF2. (A) Putative protein‐binding partners of LINC00707 were predicted by the ENCORI database (https://starbase.sysu.edu.cn/index.php). (B) A LINC00707 pull‐down was conducted, and subsequent western blot analysis revealed the interaction between LINC00707 and YTHDF2 in Hep3B and SNU449 cells. (C) The binding fact between LINC00707 and YTHDF2 was also verified by the RIP assay in Hep3B and SNU449 cells. (D) The subcellular localization of LINC00707 and YTHDF2 in Hep3B and SNU449 cells were presented by confocal images. (E) YTHDF2 mRNA expression in Sh‐NC, Sh‐LINC00707, OE‐NC and OE‐LINC00707 transfected Hep3B and SNU449 cells was measured by qRT‐PCR. (F) Analysis via western blot was performed on YTHDF2 within transfected Hep3B cells that were subjected to Chx treatment for the specified duration. (G) Analysis via western blot was performed on YTHDF2 within transfected Hep3B cells that were subjected to MG132 treatment for the specified duration. (H) Analysis via western blot was performed on YTHDF2 within Sh‐NC or Sh‐YTHDF2 Hep3B and SNU449 cells. (I) Immunoprecipitation using an anti‐Flag antibody was utilized to detect ubiquitinated YTHDF2 in transfected Hep3B cells. The experiments were performed three times. ***p < 0.001.

To unravel the potential molecular mechanisms through which LINC00707 modulates the tumorigenic properties of HCC, we employed bioinformatic analysis utilizing the ENCORI database (https://starbase.sysu.edu.cn/index.php). This analysis identified YTH N6‐methyladenosine RNA‐binding protein 2 (YTHDF2) as a promising target of LINC00707 (Figure 3A). Subsequently, we verified the interaction between LINC00707 and YTHDF2 through a combination of RNA pull‐down and subsequent western blot analysis. The results demonstrated that biotin‐labelled LINC00707 exhibited a significant pull‐down of YTHDF2 compared to the anti‐sense control (Figure 3B). Additionally, RNA immunoprecipitation (RIP) assays were conducted, revealing that the anti‐YTHDF2 antibody effectively precipitated substantial amounts of LINC00707 in comparison to the IgG control (Figure 3C). The co‐localization of LINC00707 with YTHDF2 in the cytoplasm of Hep3B and SNU449 cells was further validated using confocal microscopy (Figure 3D). These findings collectively confirm the established interaction between LINC00707 and YTHDF2.

### 3.4 LINC00707 increases the ubiquitination level of YTHDF2

Upon conducting qRT‐PCR analysis on Hep3B and SNU449 cells with manipulated LINC00707 expression, it was discerned that alterations in LINC00707 expression did not affect the levels of YTHDF2 mRNA (Figure 3E). This observation prompted the formulation of the hypothesis that LINC00707 might exert control over YTHDF2 levels through post‐translational modifications. To delve further into this hypothesis, we intervened in protein synthesis and proteasomal degradation in Hep3B cells using cycloheximide (CHX) or MG132, respectively. As illustrated in Figure 3F, in the presence of CHX, the expression of YTHDF2 protein significantly increased in LINC00707‐depleted Hep3B cells, while it remained unchanged in control Hep3B cells. Conversely, after treatment with MG132, YTHDF2 protein levels exhibited stability in both LINC00707‐depleted and control Hep3B cells. These findings suggested that LINC00707 did not influence the synthesis of the YTHDF2 protein (Figure 3G). Subsequent western blot assays conducted on LINC00707‐downregulated Hep3B and SNU449 cells indicated a negative regulation of YTHDF2 expression by LINC00707 (Figure 3H). To investigate whether LINC00707 played a role in regulating the ubiquitination of YTHDF2, an investigation involved the co‐expression of Flag‐YTHDF2 and HA‐ubiquitin in both LINC00707‐depleted and LINC00707‐overexpressed Hep3B cells. After immunoprecipitating YTHDF2 from Hep3B cells that had been treated with MG132, a significant finding emerged: in LINC00707‐overexpressed cells, YTHDF2 exhibited pronounced ubiquitination compared to control cells. Interestingly, in contrast, the introduction of LINC00707 overexpression led to a reduction in ubiquitination levels in Hep3B cells (see Figure 3I). Collectively, these findings indicate that LINC00707 mitigates the protein levels of YTHDF2 by impeding ubiquitination‐triggered protein degradation.

### 3.5 YTHDF2 overexpression aggravates NK cell‐mediated cytotoxicity

**FIGURE 4:**
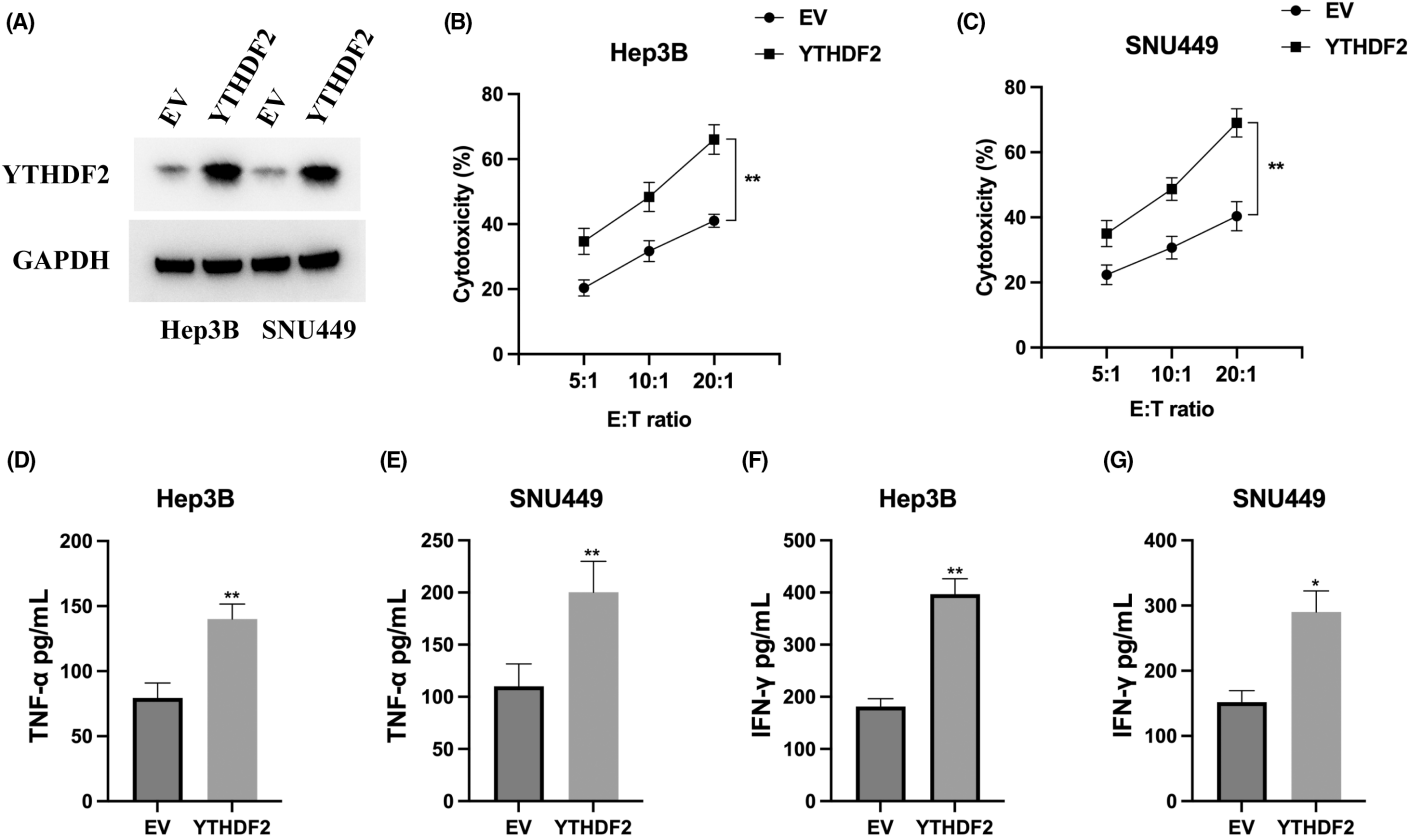
YTHDF2 overexpression aggravates NK cell‐mediated cytotoxicity. (A) Analysis via western blot was performed on Hep3B and SNU449 cells transfected with the Empty Vector (EV) and YTHDF2 vector. (B, C) The LDH assay was employed to identify the cytotoxicity of NK‐92MI cells after co‐culturing with Hep3B (B) or SNU449 (C) cells overexpressing YTHDF2, using effect‐to‐target ratios of 5:1, 10:1 and 20:1. (D, E) ELISA was used to measure the protein levels of TNF‐α in the supernatant of NK‐92MI cells that were co‐cultured with Hep3B (D) or SNU449 (E) cells overexpressing YTHDF2. (F, G) ELISA was used to measure the protein levels of IFN‐γ in the supernatant of NK‐92MI cells that were co‐cultured with Hep3B (D) or SNU449 (E) cells overexpressing YTHDF2. The experiments were performed three times. *p < 0.05, **p < 0.01.

The connection between YTHDF2 and NK cell‐mediated cytotoxicity has been previously demonstrated.^14^ To further explore the role of YTHDF2 in NK cell function in the context of HCC, we implemented a strategy to upregulate YTHDF2 expression in Hep3B and SNU449 cells, and the successful upregulation was confirmed through western blot analysis (Figure 4A). Subsequently, we evaluated the cytotoxic effect of NK cells on HCC cells using an LDH release assay. Interestingly, in the group of HCC cells overexpressing YTHDF2, we observed a significant enhancement in the cytotoxicity exerted by NK‐92MI cells compared to the Empty Vector (EV) group (Figure 4B,C). Furthermore, we explored whether the increased expression of YTHDF2 could stimulate the secretion of cytokines by NK‐92MI cells. We measured the levels of secreted TNF‐α and IFN‐γ in the supernatant of NK‐92MI cells co‐cultured with HCC cells overexpressing YTHDF2 using ELISA (Figure 4D–G). The results indicated that the heightened expression of YTHDF2 resulted in an intensified release of cytokines by NK‐92MI cells, thereby amplifying their cytotoxic efficacy.

### 3.6 YTHDF2 overexpression inhibits HCC cell proliferation, migration and invasion

**FIGURE 5:**
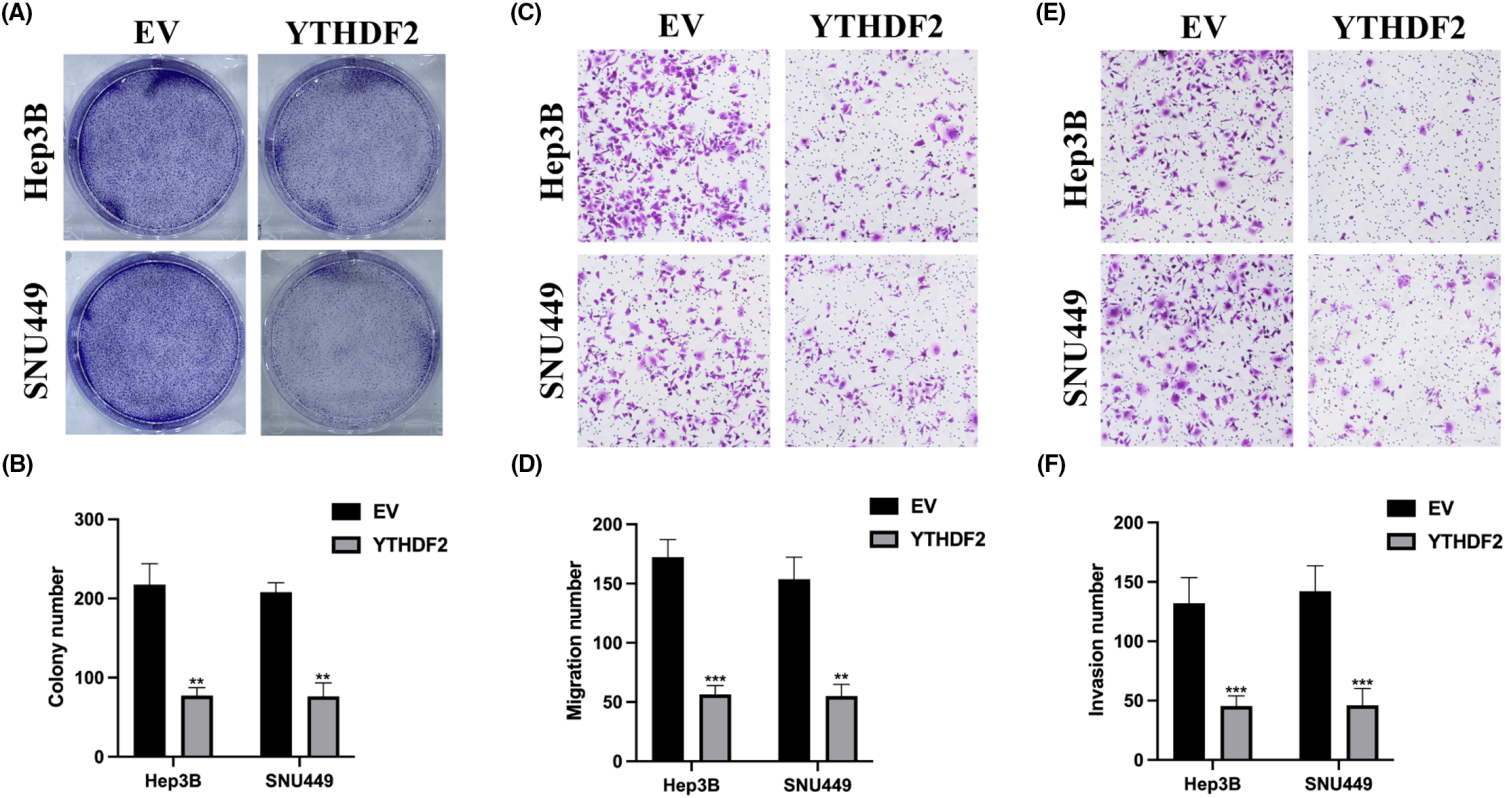
YTHDF2 overexpression inhibits hepatocellular carcinoma cell proliferation, migration and invasion. (A, B) Cell colony assay was conducted to evaluate cell proliferation level. (C, D) Transwell migration assay was used to detect cell migration ability. (E, F) Transwell invasion assay was utilized to elucidate cell invasion level. The experiments were performed three times. **p < 0.01, ***p < 0.001.

We conducted cell colony and transwell assays to assess the influence of YTHDF3 on various aspects of HCC cell behaviour, including proliferation, migration and invasion. As illustrated in Figure 5A,B, the overexpression of YTHDF2 led to a reduction in the proliferation capacity of HCC cells. Likewise, YTHDF2 overexpression curbed the migratory potential of HCC cells, as evidenced in Figure 5C,D. Additionally, YTHDF2 overexpression also restrained the invasive behaviour of HCC cells, as indicated in Figure 5E,F. These findings collectively suggest that YTHDF2 plays a pivotal role in HCC progression by influencing NK‐92MI cell cytotoxicity and modulating the tumorigenic characteristics of HCC cells.

### 3.7 LINC00707 inhibits NK cell‐mediated cytotoxicity through regulating YTHDF2

**FIGURE 6:**
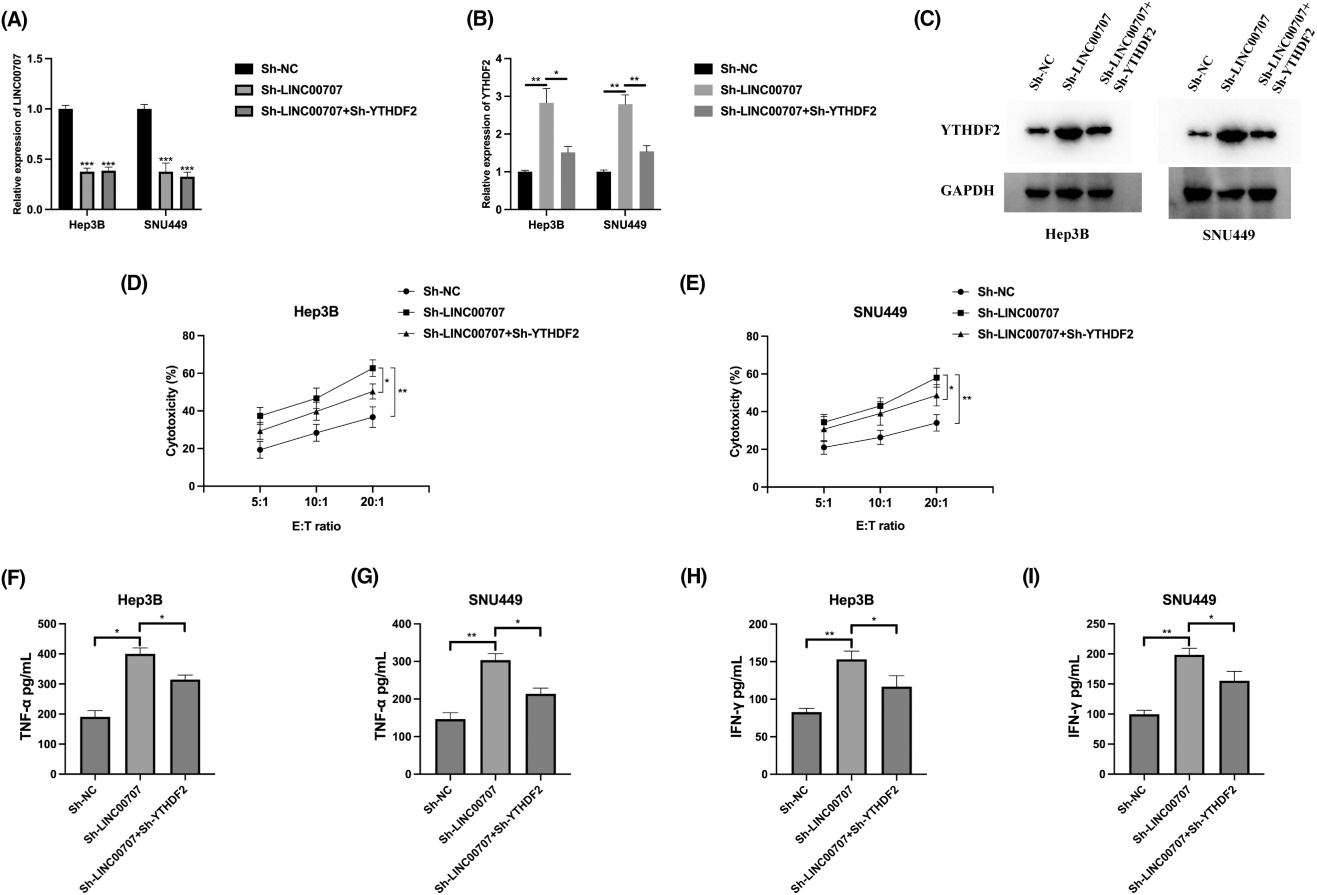
LINC00707 inhibits NK cell‐mediated cytotoxicity through decreasing YTHDF2 stability. (A) Analysis via qRT‐PCR was performed on Hep3B or SNU449 cells, transfected with Sh‐NC, Sh‐LINC00707 and Sh‐LINC00707+Sh‐YTHDF2, to measure LINC00707 expression. (B) Analysis via qRT‐PCR was performed on transfected Hep3B or SNU449 cells to measure YTHDF2 mRNA expression. (C) Analysis via western blot was performed on transfected Hep3B or SNU449 cells to measure YTHDF2 protein expression. (D, E) The LDH assay was employed to identify the cytotoxicity of NK‐92MI cells after co‐culturing with transfected Hep3B (D) or SNU449 (E) cells, using effect‐to‐target ratios of 5:1, 10:1 and 20:1. (F, G) ELISA was used to measure the protein levels of TNF‐α in the supernatant of NK‐92MI cells that were co‐cultured with transfected Hep3B (F) or SNU449 (G) cells. (H, I) ELISA was used to measure the protein levels of IFN‐γ in the supernatant of NK‐92MI cells that were co‐cultured with transfected Hep3B (H) or SNU449 (I) cells. The experiments were performed three times. *p < 0.05, **p < 0.01, ***p < 0.001.

We conducted investigations to ascertain whether LINC00707 contributes to the progression of HCC by modulating YTHDF2 expression. Hep3B and SNU449 cells were transfected with Sh‐NC, Sh‐LINC00707 and Sh‐LINC00707+Sh‐YTHDF2, and the outcomes were evaluated using qRT‐PCR and western blot assays (Figure 6A–C). Initially, we assessed the impact of NK‐92MI cell cytotoxicity on Hep3B and SNU449 cells. As depicted in Figure 6D,E, the depletion of LINC00707 significantly enhanced NK‐92MI cell cytotoxicity against Hep3B and SNU449 cells, an effect that was mitigated upon co‐transfection with Sh‐YTHDF2. Furthermore, through ELISA analysis of the cell supernatant, we quantified the levels of secreted TNF‐α and IFN‐γ. The reduction of LINC00707 led to an increase in cytokine release by NK‐92MI cells, and this effect could be reversed by Sh‐YTHDF2 (refer to Figure 6F–I).

### 3.8 YTHDF2 is responsible for the role of LINC00707 in HCC tumorigenic progression

**FIGURE 7:**
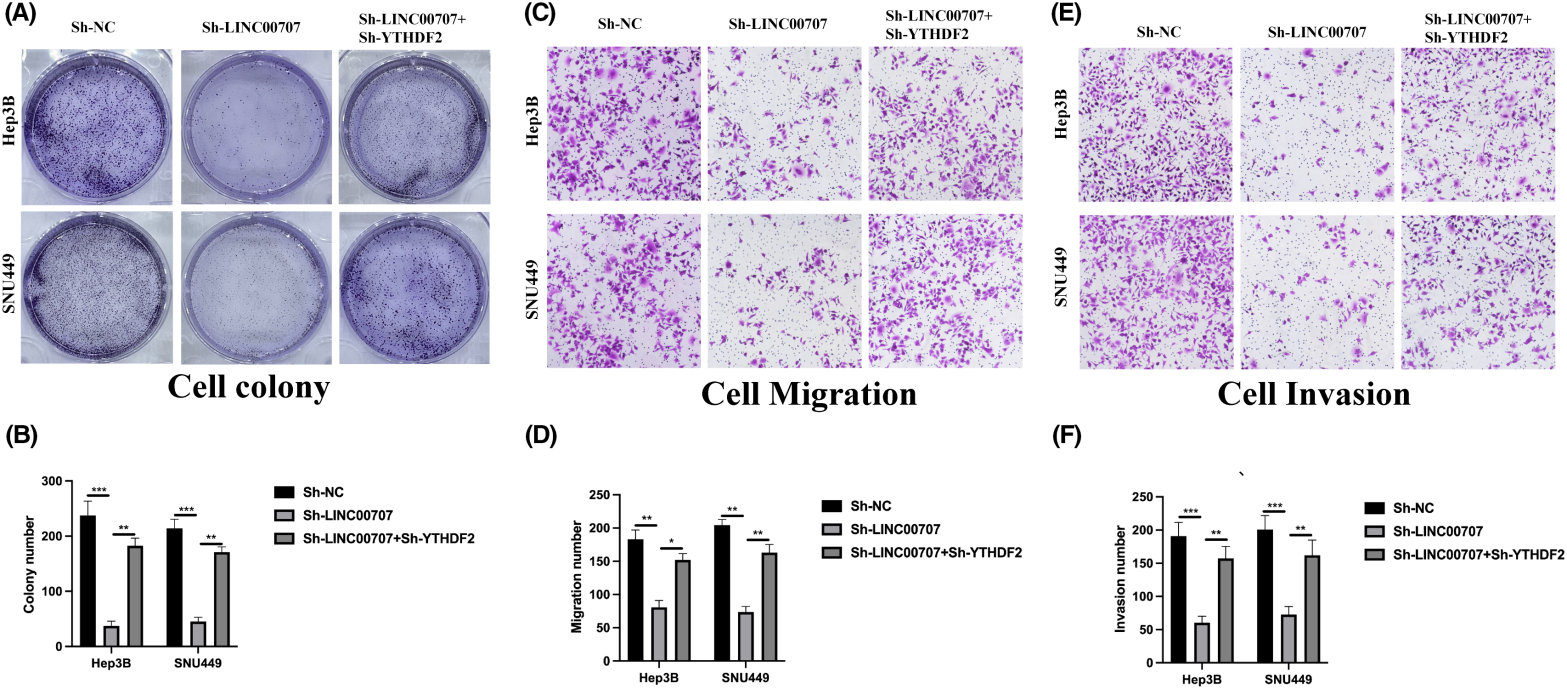
YTHDF2 is responsible for the role of LINC00707 in hepatocellular carcinoma tumorigenic progression. (A, B) Cell colony assay was conducted to evaluate cell proliferation level. (C, D) Transwell migration assay was used to detect cell migration ability. (E, F) Transwell invasion assay was utilized to elucidate cell invasion level. The experiments were performed three times. **p < 0.01, ***p < 0.001.

We conducted investigations into the effects of the LINC00707/YTHDF2 axis on HCC cell behaviours. As depicted in Figure 7A,B, the reduction in HCC cell proliferation resulting from LINC00707 downregulation could be alleviated through the simultaneous knockdown of YTHDF2. This trend was similarly observed in transwell migration assays (Figure 7C,D) as well as invasion assays (Figure 7E,F). These results suggest that LINC00707 plays a role in the progression of HCC by modulating YTHDF2 expression. Additionally, the influence of the LINC00707/YTHDF2 axis on NK cells may partially explain the impact of LINC00707 on the tumorigenic properties of HCC.

## 4 DISCUSSION

HCC stands out as a prevalent gastrointestinal malignancy, with considerable unexplored territory in terms of understanding the fundamental pathological mechanisms driving its onset and metastasis.^15^, ^16^ In the recent past, lncRNAs have emerged as crucial players in orchestrating the progression of diverse tumours through their interactions with proteins or nucleic acids.^17^, ^18^, ^19^ This study aimed to identify a functionally relevant lncRNA in the progression of HCC and elucidate the molecular mechanisms governing its actions. Intriguingly, an examination of lncRNA microarrays involving three sets of HCC tissues unveiled LINC00707 as one of the significantly upregulated lncRNAs in HCC tissues. LINC00707, a recently discovered lncRNA, has versatile regulatory roles in different diseases. It exerts anti‐inflammatory and anti‐apoptotic effects by interacting with miR‐30a‐5p and influences osteogenic differentiation through the modulation of miR‐103a‐3p.^20^, ^21^ In the context of autoimmune diseases, its suppression aligns with protective mechanisms against myocardial injury, particularly through the inhibition of miR‐23.^22^ The dynamic interplay between LINC00707, microRNAs and target genes underscores its potential as a therapeutic target in various pathological conditions.^23^, ^24^ Although some investigations have shed light on the role of LINC00707 in HCC,^12^, ^13^ there remains a substantial gap in our understanding. As a result, this investigation validated the oncogenic role of LINC00707 in HCC through assessments like cell colony assays, transwell migration and transwell invasion assays. Further exploration into the underlying mechanisms involved bioinformatic analyses, highlighting YTHDF2 as a potential binding protein for LINC00707.

YTHDF2 stands out due to its distinctive ability to facilitate the degradation of RNA molecules modified with m6A, a function that sets it apart from other members of its protein family.^25^ Notably, serving as an m6A reader, YTHDF2 is recognized for its role in identifying and transporting m6A‐modified RNAs to processing bodies, subsequently expediting their degradation.^25^ Furthermore, emerging research has shed light on YTHDF2's close association with inflammation and the polarization of macrophages.^26^, ^27^ Additionally, YTHDF2 has emerged as a promising diagnostic, immunotherapeutic and prognostic biomarker for a range of immune‐related disorders.^28^, ^29^ Emerging research has highlighted the potentially dual role of YTHDF2 in both the initiation and aggressive progression of HCC. On one hand, YTHDF2 has been shown to support the phenotype of liver cancer stem cells and facilitate metastasis by enhancing the stability of m6A modifications on OCT4 transcripts.^30^ Conversely, YTHDF2 also functions as a tumour suppressor by hastening the degradation of EGFR transcripts,^31^ which in turn curtails HCC growth. This apparent paradox emphasizes the necessity for more thorough investigations to accurately elucidate the specific roles and underlying mechanisms associated with the m6A reader YTHDF2 in the context of HCC.

The results of our investigation unveiled a substantial interplay between LINC00707 and YTHDF2 within HCC cells. Specifically, LINC00707 was found to hinder the expression of the YTHDF2 protein in HCC cells, achieved through the facilitation of ubiquitination‐mediated degradation of YTHDF2. Consequently, it was demonstrated that YTHDF2 plays a dual role in HCC: it enhances the cytotoxicity of NK‐92MI cells against HCC cells and functions as an oncogene in HCC progression by promoting cell proliferation, migration and invasion. Importantly, the influence of LINC00707 on HCC advancement arises from its capacity to regulate the expression of YTHDF2. These findings introduce a novel perspective on the roles of LINC00707 and YTHDF2 in HCC, enriching our understanding in this domain.

## AUTHOR CONTRIBUTIONS

**Weiming Wei:** Data curation (equal); formal analysis (equal); investigation (equal); software (equal); validation (equal); visualization (equal). **Libai Lu:** Data curation (equal); software (equal); validation (equal); visualization (equal). **Jiasheng Ma:** Methodology (equal); visualization (equal). **Zongjiang Luo:** Data curation (equal); visualization (equal). **Xijuan Tan:** Investigation (equal); validation (equal). **Jianchu Wang:** Conceptualization (equal); investigation (equal); methodology (equal); project administration (equal); resources (equal); supervision (equal); writing – original draft (equal).

## FUNDING INFORMATION

This study was supported by the National Natural Science Foundation of China (No. 82060441 to JC Wang).

## CONFLICT OF INTEREST STATEMENT

The authors affirm that they do not have any identifiable financial conflicts of interest or personal relationships that could have been perceived as potentially influencing the research presented in this paper.

## Data Availability

The data used to support the findings of this study are available from the corresponding author upon request.
